# Low heart deceleration capacity imply higher atrial fibrillation-free rate after ablation

**DOI:** 10.1038/s41598-018-23970-7

**Published:** 2018-04-03

**Authors:** Zifan Chen, Yichen Yang, Cao Zou, Yunyun Zhang, Xingmei Huang, Xun Li, Xiangjun Yang

**Affiliations:** 1 Departmentof Cardiology, the First Affiliated Hospital of Soochow University, 296 Shizi Street, Suzhou, 215006 China; 2 Departmentof Electrocardiography, the First Affiliated Hospital of Soochow University, 296 Shizi Street, Suzhou, 215006 China; 3 Departmentof Echocardiography, the First Affiliated Hospital of Soochow University, 296 Shizi Street, Suzhou, 215006 China

## Abstract

How deceleration capacity (DC) and acceleration capacity (AC) of heart rate associated with atrial fibrillation (AF) and ablation is still not clear. The dynamic changes of AC, DC and conventional heart rate variability (HRV) parameters were characterized in 154 subjects before circumferential pulmonary veins isolation (CPVI) and three days, 3 months and 6 months after CPVI. The DCs of the recurrent group decreased significantly at each time point after CPVI; the DCs of the recurrence-free group before CPVI and three days, 3 months and 6 months after CPVI were 7.06 ± 1.77, 3.79 ± 1.18, 4.22 ± 1.96 and 3.97 ± 0.98 ms respectively, which also decreased significantly at each time point and were significantly lower than these of recurrent group. Conversely, the AC of recurrent and recurrence-free groups increased significantly at each time point after CPVI; the ACs of recurrence-fee group were significantly higher than these of recurrent group at each time point. No stable difference trend of HRV parameters was found between two groups. Further Kaplan–Meier analysis showed that DC < 4.8 ms or AC ≥ −5.1 ms displayed significant higher recurrence-free rates. In conclusion, high AC and low DC imply higher AF-free rate after ablation.

## Introduction

The heart is richly innervated by the sympathetic and parasympathetic (vagal) nerves^1–3^. Normal autonomic nervous system (ANS) coordination is critical for cardiac function maintaining^1–3^. However, disorganized ANS activation can induce atrial fibrillation (AF)^1–4^. The overall role of sympathetic nerves in the heart is to modulate the automaticity of sinoatrial node and the conductivity of atrioventricular node^1–3^. Abnormal adrenergic activation may promote focal activity of atrial myocytes via enhanced automaticity, early afterdepolarization or delayed afterdepolarization; which may act as a trigger of AF or an AF-maintaining driver^1–3^. The general effect of vagal nerve on the heart is to reduce the automaticity of sinoatrial node and the conductivity of atrioventricular node^1–3^. Abnormal parasympathetic activation produces spatially heterogeneous action potential and refractory period abbreviation, which may promote the occurrence and maintenance of re-entrant activity^3^.

Recent basic science concepts and clinical catheter-based AF-ablation techniques showed that most paroxysmal AF is triggered by ectopic firing originating from the cardiomyocyte sleeves extending to the pulmonary vein (PV) and the junction between PV and left atrium^5–9^. Thus, PV isolation (PVI) rapidly replaced focal ablation and emerged as a main therapeutic strategy to treat AF^9–12^. The efficacy and major complications of catheter ablations are now approximately 70–80% and 5% respectively^5,8,9^.

Despite encouraging achievements of catheter ablation in AF therapy, how to improve the long-term efficacy requires further knowledge on how ablation rectified the ANS modulation in cardiac electrophysiology^5,9^. Current measures of cardiac electrophysiology, including conventional heart rate variability (HRV), are hard to distinguish the respective role of sympathetic and parasympathetic nerves in the cardiac pathophysiology of AF^13,14^. In 2006, Baver *et al*. created a series of signal processing technology and algorithm to separately characterize the deceleration and acceleration capacities of the heart rate using 24-h ambulatory electrocardiogram^15^. Their calculation principle and later reports suggested that AC and DC might distinguish and quantify between vagal and sympathetic nervous system roles that affect cardiac electrophysiology^15–17^.

In this report, to evaluate whether AC/DC have the potential to discriminate AF recurrence, 154 qualified paroxysmal AF subjects were assembled. The dynamic change trend of AC and DC before and after CPVI was compared between recurrent and recurrence-free subjects.

## Patients and Methods

### Ethical issues

The review board of the First Affiliated Hospital of Soochow University approved this protocol in accordance with the ethical standards of the relevant national and institutional committees on human experimentation and with the Helsinki Declaration of 1975, as revised in 2008. Written informed consent regarding procedures and medical data were obtained from all of the patients according to the guidelines of the Chinese National Ethics Regulation Committee. All patients were informed of their rights to withdraw consent personally or via kin, caretakers, or guardians.

### Subjects and preoperative preparation

All subjects involved in this study were drug-resistant paroxysmal AF patients. Paroxysmal AF is defined as an episode of AF that terminates spontaneously or with intervention within 7 days of onset or episodes may recur with variable frequency^3^. Drug-resistant paroxysmal AF was defined at least 2 ineffective antiarrhythmic drugs in suppressing AF recurrence^3^.

Initially, 219 patients with paroxysmal AF were treated with radiofrequency ablation in our Department of Cardiology from January 2013 to November 2015 (For details, see results). 24 hours before CPVI, transesophageal echocardiography was performed to exclude the possibility of thrombus in left atrial appendage and left atrium, the anatomical structures of the left atrium and pulmonary vein were determined by CT scan. Patients with a previous catheter ablation; coronary insufficiency; recent myocardial infarction; clinical symptoms of heart failure; relevant valvular dysfunction; terminal renal insufficiency, obstructive sleep apnea, chronic obstructive pulmonary disease or any other relevant pulmonary disease; hyperthyroidism/hypothyroidism; inflammatory diseases; diabetes and sick sinus syndrome were excluded from this study.

### Circumferential pulmonary vein isolation (CPVI)

Under the guidance of three-dimensional mapping system (EnSite 3000, St. Jude Medical Inc., Little Canada, MN), CPVI was performed using cold saline perfusion (flow rate 17 mL/min, 30–35 Watts). Under local anesthesia; puncture the left femoral vein; insert 10 polar sinus electrodes to the distal coronary vein and fix with skin paste; puncture the right femoral vein twice; insert the Agilis and SL1 long sheaths (St. Jude Medical Inc., Little Canada, MN) respectively. The puncture needle (St. Jude Medical Inc., Little Canada, MN) is sent to the atrial septum via the SL1 long sheath; SL1 long sheath are sent into the left atrium after piercing atrial septum. The electrodes of saline infusion radiofrequency catheter ablation are sent to left atrium via the Agilis long sheath. Under the positioning of the EnsiteNavx (St. Jude Medical Inc., Little Canada, MN), complete the following procedures: construction of the three-dimensional model of left atrium; mapping the potentials of the left superior, left inferior, right superior and right inferior pulmonary veins; determination of pulmonary vein orifice and left atrial appendage position; setting the circumferential pulmonary vein ablation line; loading the electrodes of saline infusion radiofrequency catheter ablation; the left and right pulmonary vein macrocyclic isolation along the ablation line. Dormant conduction was assessed by isoproterenol infusion, pulmonary vein pacing, coronary vein and intra atrial pacing.

### Postoperative treatment and follow-up

All patients received postoperative dabigatran (110 mg, bid) for anticoagulation; amiodarone (0.2 g, qd) to maintain sinus rhythm; and omeprazole (20 mg, qd) to suppress gastric acid for 3 months. Patients with discomfort will be submitted for the ECG monitoring. The cardiac electrophysiology and echocardiography of all patients will be evaluated using 24-h ambulatory electrocardiograms and a Sonos 5500 type ultrasound machine (Philips, Best, Netherlands) three days, 3 months and 6 months after operation to learn the efficacy and complications of CPVI.

### Definition of postoperative recurrence of AF

The time point to define postoperative recurrence of AF has not yet unified. Three months after radiofrequency ablation is a blank period^3^. According to the “2017 HRS/EHRA/ECAS/APHRS/SOLAECE expert consensus statement on catheter and surgical ablation of atrial fibrillation”, the recurrence of AF within 3 months was defined as early recurrence^3^. In this study, the average time of the third time point (3 months after CPVI) was 90 ± 5 days, so, paroxysmal atrial arrhythmias; such as atrial tachycardia, atrial flutter and AF lasting more than 30 seconds detected at this time point was defined as early recurrence. AF recurrence > 30 s recorded at the fourth time point (6 months after CPVI) was defined as late recurrence.

### Calculation of DC and AC using 24 h ambulatory electrocardiograms

To monitor the cardiac electrophysiology of all participates before and after ablation, a Holter monitor test was performed using a Holter Monitoring DigiTrak XT Holter System (Philips, Best, Netherlands).

A phase-rectified signal averaging (PRSA) algorithm^17^ to the RR interval series, which is capable of detecting and quantifying quasi-periodic oscillations masked by nonperiodic components, artifacts and ectopic beats, was adopted in analysis of 24-h ambulatory electrocardiograms. The DC and AC were computed with timescales (*T*) = 1 and wavelet scales (*s*) = 2. To avoid errors caused by artifacts and ectopic rhythm; abnormal RR intervals, defined as RR intervals that change by more than 20% from the previous RR interval, were removed automatically; sinus arrhythmia and ventricular rhythm were further excluded manually according to the P waves morphology and QRS waves shape respectively.

Briefly, step 1, Definition of decelerating and accelerating anchors: heartbeat intervals longer than the preceding interval were identified as decelerating anchors; heartbeat intervals shorter than the preceding interval were identified as accelerating anchors. Step 2, definition of cardiac electrical segments: segments of interval data around the decelerating and accelerating anchors are selected. Step 3, phase rectification: the cardiac electrical segments described above were aligned at the decelerating and accelerating anchors. Step 4, signal averaging: the phase-rectified signal averaging signal X(i) was obtained by averaging the signals within the aligned cardiac electrical segments. Step 5, quantification of deceleration and acceleration capacities using the following formula: DC (AC) = [X(0) + X(1) − X(−1) − X(−2)]/4.

### HRV measure and analysis

According to the guidelines: Heart rate variability, Standards of measurement, physiological interpretation, and clinical use^14^, the HRV parameters were computed using same set of 24 h ambulatory electrocardiograms while calculating AC and DC. The quality control of the RR interval series was performed as AC/DC computation, that is, abnormal RR intervals, defined as RR intervals that change by more than 20% from the previous RR interval, were removed automatically; sinus arrhythmia and ventricular rhythm were further excluded manually according to the P waves morphology and QRS waves shape respectively. Indices of time-domain methods include the full-course standard deviation of NN (NN is used in place of RR to emphasize that the processed beats are normal beats) intervals (SDNN); root mean square of successive differences (RMSSD, which refers to the square root of the mean of the squares of the successive differences between adjacent NNs); indices of frequency-domain methods, which assign bands of frequency and then count the number of NN intervals that match each band, these include high frequency (HF), low frequency (LF) and the LF/HF; and average heart rate; were computed using Kubios HRV analysis software (http://kubios.uef.fi).

### Echocardiography

As previously described^16^, a transthoracic echocardiographic examination was performed using a Sonos 5500 type Ultrasound machine (Philips, Best, Netherlands) with a 2.5 Hz transducer. The parameters measured with the M-mode technique included left ventricular end-diastolic, end-systolic and left atrial diameters. The measurement of the left ventricular ejection fraction was performed using the Simpson’s biplane method.

All of the echocardiographic and above cardiac electrophysiological examinations were performed and analyzed licensed technicians who were blinded to clinical data and group division.

### Statistical analysis

Continuous variables are presented as means ± standard deviation (SD); a non-paired Student’s t-test were used for comparison between groups. Categorical data are summarized as frequencies and percentages and compared using chi-square test and/or Fisher’s exact test. Kaplan–Meier analyses with log-rank tests were used to calculate AF recurrence-free survival over time and compare recurrence rates between groups. The performance of discrimination was evaluated by an area under receiver operating characteristic (ROC) curve (AUC). P values less than 0.05 (two-sided) were deemed statistically significant. Statistical analyses were performed using SPSS version 17.0 (SPSS Inc., Chicago, IL, USA).

## Results

### Participants

**Figure 1:**
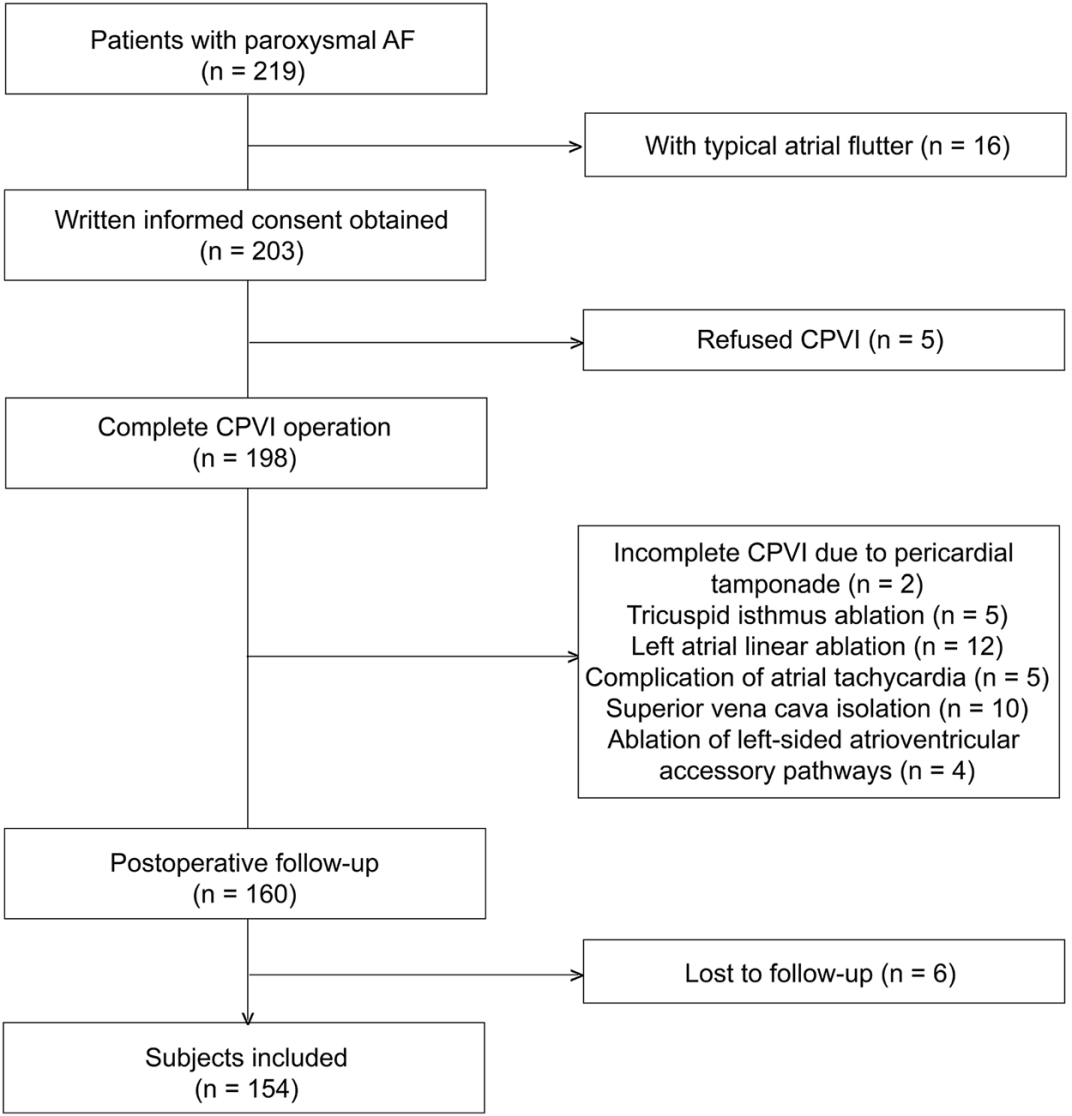
Study flowchart of patient selection. AF, atrial fibrillation; CPVI, circumferential pulmonary vein isolation.

219 patients with drug refractory paroxysmal AF were collected initially from January 2013 to November 2015. Of whom, 16, 5, 2, 5, 12, 5, 10, 4 and 6 individuals were excluded from this reports due to typical atrial flutter, refusing ablation, incomplete CPVI due to pericardial tamponade, tricuspid isthmus ablation, left atrial linear ablation, complication of atrial tachycardia, superior vena cava isolation, ablation of left-sided atrioventricular accessory pathways and lost to follow-up, respectively. Finally, 154 qualified patients with paroxysmal AF were involved in this study (Fig. 1). Of the 154 participants; their average age was 60.0 ± 10.1 years old; 94 (61.0%) and 60 (39.0%) were males and females; the mean PAF duration was 3.5 ± 6.0 years; 86 (55.8%) and 7 (4.6%), complicated with hyptertension and CHD respectively; 85 (55.2%), 26 (16.9%) and 74 (48.1%) were treated with aminodarone, propafenone and metoprolol respectively; the DC, AC, AHR, SDNN, RMSSD and LF/HF were 7.15 ± 2.15 ms, −7.46 ± 2.21 ms, 66.6 ± 13.0 bpm, 144.1 ± 55.5 ms, 38.2 ± 23.9 ms and 1.76 ± 1.33 respectively; the LAd and LVEF were 40.3 ± 5.6 mm and 0.68 ± 0.06 respectively (Table 1).

**Table 1:** Characteristics of the subjects (n = 154).

Demography	
Age (yrs)	60.0 ± 10.1
Male (%)	94 (61.0)
**History**	
PAF duration (yrs)	3.5 ± 6.0
Complication	
Hypretension, case (%)	86 (55.8)
CHD, case (%)	7 (4.6)
**Treatment**	
Amiodarone, case (%)	85 (55.2)
Propafenone, case (%)	26 (16.9)
Metoprolol, case (%)	74 (48.1)
**Electrophysiology**	
DC (ms)	7.15 ± 2.15
AC (ms)	−7.46 ± 2.21
AHR (bpm)	66.6 ± 13.0
SDNN (ms)	144.1 ± 55.5
RMSSD (ms)	38.2 ± 23.9
LF/HF	1.76 ± 1.33
**Echocardiography**	
LAd (mm)	40.3 ± 5.6
LVEF	0.68 ± 0.06

Continuous variables were presented as means ± standard deviation (SD), and categorical data were presented as the number (percentage). Abbreviations: PAF, paroxysmal atrial fibrillation; CHD, coronary heart disease; AC, acceleration capacity; DC, deceleration capacity; AHR, average heart rate; SDANN, standard deviation of the averages of NN intervals in all 5-min segments of the entire recording; RMSSD, root mean square of successive differences; HF, high frequency; LF, low frequency; LAd, left atrial diameter; LVEF, left ventricular ejection fraction.

### AC, DC and HRV changes in AF patients before and after CPVI

**Figure 2:**
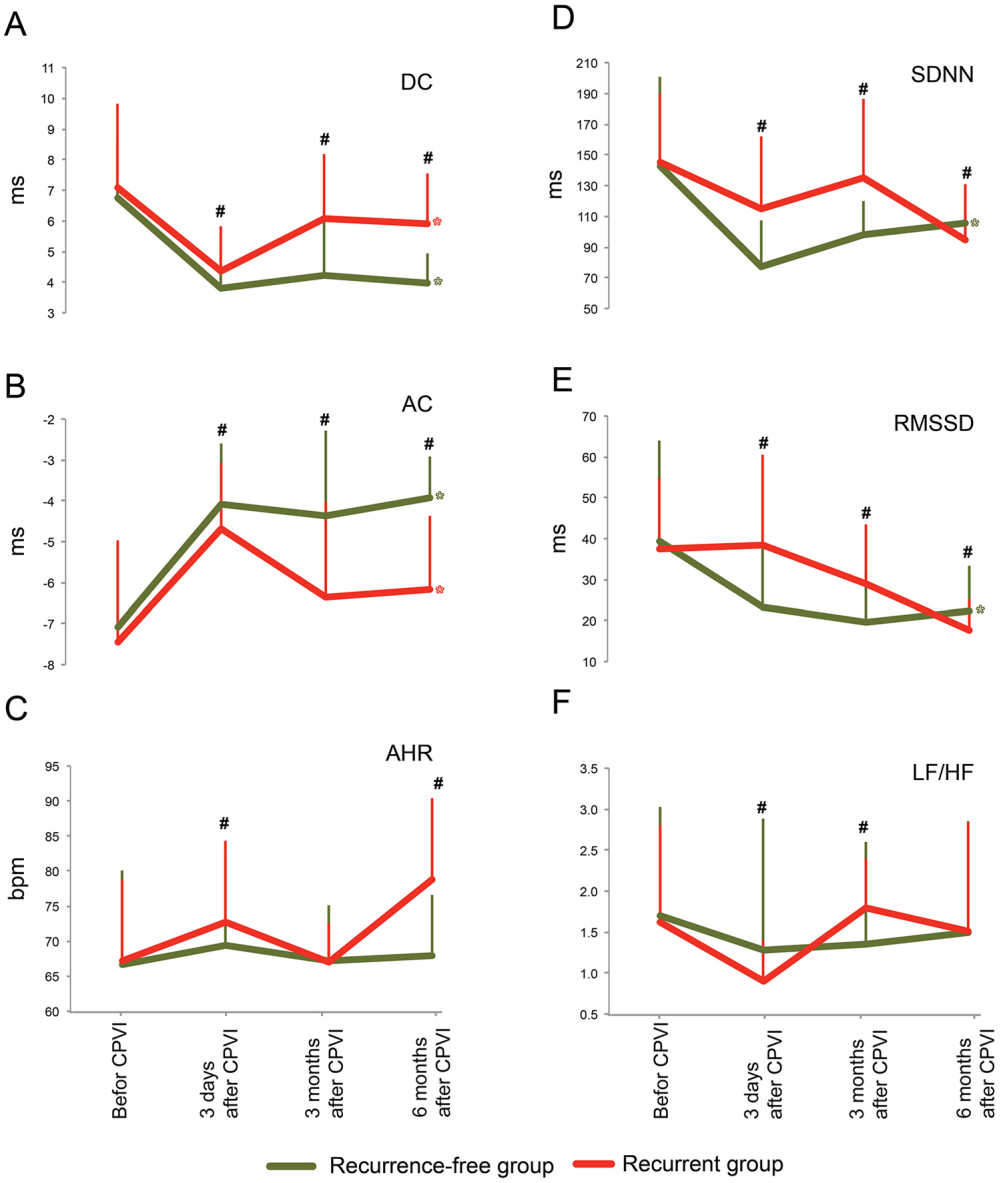
The change tendency of each index before and after CPVI of the recurrence-free and recurrence groups. *indicates the significance at each time point after CPVI when compared with that before CPVI. #indicates the significance at a time point between recurrence-free group and recurrence group. For abbreviation, please see Table 1 or Table 2.

First, let’s look at DC: the DCs of the recurrent group before CPVI and three days, 3 months and 6 months after CPVI were 7.39 ± 2.90, 4.36 ± 1.46, 6.08 ± 2.09 and 5.89 ± 1.65 ms respectively; the DC decreased significantly at three days after CPVI; although DCs elevated 3 and 6 months after CPVI, it kept still significantly lower than that before CPVI (Fig. 2A). The DCs of the recurrence-free group before CPVI and three days, 3 months and 6 months after CPVI were 7.06 ± 1.77, 3.79 ± 1.18, 4.22 ± 1.96 and 3.97 ± 0.98 ms respectively; the DC also decreased significantly three days after CPVI and kept at relatively low level through out 6 months; impressively, the DCs were significantly lower in recurrence-free group than these in recurrent group at each time point (Fig. 2A).

Next, let’s look at AC: the ACs of the recurrent group before CPVI and three days, 3 months and 6 months after CPVI were −7.78 ± 2.76, −4.69 ± 1.62, −6.36 ± 2.34 and −6.16 ± 1.78 ms respectively; the AC increased significantly three days after CPVI; although ACs declined 3 and 6 months after CPVI, it kept still significantly higher than that before CPVI (Fig. 2B). The ACs of the recurrence-free group before CPVI and three days, 3 months and 6 months after CPVI were −7.33 ± 1.95, −4.09 ± 1.49, −4.38 ± 2.10 and −3.92 ± 1.01 ms respectively; the AC also increased significantly three days after CPVI and kept at relatively high level through out 6 months; impressively again, the DCs were significantly higher in recurrence-free group than these in recurrent group at each time point (Fig. 2B).

Then, let’s look at other indices: of the recurrent group, three days after the CPVI, the AHR was significantly higher and all others except RMSSD were significantly lower than those before CPVI; of the recurrence-free group, three days after the CPVI, the AHR was significantly higher and all others indies were significantly lower than those before CPVI (Fig. 2C–F); different from AC and DC, no stable difference trend was found between two groups, although “significant differences” emerged frequently (Fig. 2C–F).

In conclusion, AC and DC displayed a relative superiority to discriminate the recurrence-free and recurrence subjects.

### CPVI induces the AC and DC changes

To know further the changes of AC, DC and the differences of these indices between recurrence-free and recurrent groups were caused by ablation, the baseline data of recurrence-free and recurrence groups were compared. As showed in Table 2, before the ablation, no significant difference between two groups, including disease history, complications, treatment, electrophysiology and echocardiography was found. As Amiodarone, propafenone and metoprolol administration might alter the electrocardiographic activity. The effect of these medicines on DC and AC was further assessed by comparing the mean AC and DC of treated and untreated subjects. As showed in Table 3, none of the three medicines changed the HRV indexes or AC and DC significantly. The above analyses suggest that CPVI induces the AC and DC changes.

**Table 2:** Baseline data of recurrence-free and early recurrent groups before CPVI.

Indies	Recurrence-free group(n = 100)	Early recurrence group(n = 54)	*P*
**Demography**			
Age (yrs)	59.8 ± 9.8	60.2 ± 10.7	*0.848*
Sex, M/F	61/39	33/21	*0.845*
History			
PAF duration (yrs)	3.1 ± 5.0	4.2 ± 7.5	*0.272*
**Complication**			
Hypretension, case (%)	56 (56.0)	30 (55.6)	*0.920*
CHD, case (%)	4 (4.0)	3 (5.5)	*0.689*
**Treatment**			
Amiodarone, case (%)	51 (51.0)	34 (63.0)	*0.118*
Propafenone, case (%)	17 (17.0)	9 (16.4)	*0.899*
Metoprolol, case (%)	47 (47.0)	27 (49.1)	*0.849*
**Electrophysiology**			
DC (ms)	7.06 ± 1.77	7.39 ± 2.90	*0.494*
AC (ms)	−7.33 ± 1.95	−7.78 ± 2.76	*0.367*
AHR (bpm)	65.8 ± 13.0	68.4 ± 12.9	*0.375*
SDNN (ms)	145.5 ± 60.5	140.6 ± 41.5	*0.700*
RMSSD (ms)	38.0 ± 20.7	42.1 ± 30.6	*0.438*
LF/HF	1.78 ± 1.41	1.69 ± 1.15	*0.760*
**Echocardiography**			
LAd (mm)	40.5 ± 5.6	39.9 ± 5.8	*0.654*
LVEF	0.67 ± 0.07	0.69 ± 0.05	*0.121*

Continuous variables are presented as means ± standard deviation (SD); categorical data are summarized as frequencies and percentages. Differences between groups were examined using the Student’s t test and the chi-square test according to the characteristics of the data distribution. For abbreviations, please see Table 1.

**Table 3:** Medication had not changed the HRV, AC and DC significantly.

Aminodarone			
	Treated group (N = 85)	Untreated group (N = 69)	*P*
DC (ms)	7.10 ± 2.09	7.14 ± 2.17	0.516
AC (ms)	−7.16 ± 2.01	−7.07 ± 2.11	0.187
AHR (bpm)	67.1 ± 13.2	67.4 ± 12.9	0.800
SDNN (ms)	142.5 ± 57.2	143.9 ± 58.7	0.380
RMSSD (ms)	39.2 ± 21.8	38.9 ± 22.1	0.721
LF/HF	1.71 ± 1.30	1.75 ± 1.13	0.293
**Propafenone**			
	Treated group (N = 26)	Untreated group (N = 128)	*P*
DC (ms)	7.11 ± 2.07	7.17 ± 2.07	0.385
AC (ms)	−7.14 ± 2.11	−7.17 ± 2.01	0.586
AHR (bpm)	67.8 ± 13.9	66.5 ± 11.7	0.343
SDNN (ms)	144.6 ± 58.2	143.7 ± 58.3	0.605
RMSSD (ms)	38.8 ± 21.3	38.3 ± 21.7	0.849
LF/HF	1.74 ± 1.31	1.75 ± 1.17	0.921
**Metoprolol**			
	Treated group (N = 74)	Untreated group (N = 80)	*P*
DC (ms)	7.13 ± 2.19	7.12 ± 2.27	0.272
AC (ms)	−7.13 ± 2.11	−7.17 ± 2.01	0.181
AHR (bpm)	67.8 ± 13.4	66.9 ± 12.0	0.425
SDNN (ms)	142.3 ± 57.2	143.9 ± 58.7	0.058
RMSSD (ms)	38.2 ± 20.1	38.7 ± 19.9	0.744
LF/HF	1.74 ± 1.31	1.75 ± 1.03	0.375

Data are presented as means ± SD. Differences were examined using the using the Student’s t test. The dosages of aminodarone, propafenone and metoprolol are 0.2 g, qd; 150 mg, tid; and 47.5 mg, qd; respectively. For abbreviation, please see Table 1.

### Recurrence-free rates delimited by the cut off values of AC and DC

The above data showed that AC and DC displayed certain potentials to discriminate recurrence and recurrence-free groups after ablation, we then tried to perform Kaplan–Meier analysis using cut off values of AC and DC.

Firstly, the receiver operating characteristic (ROC) curve was plotted using AC and DC values of each patient. The area under ROC curve (AUC) of AC and DC were 0.747 (95% CI 0.618 to 0.875) and 0.758 (95% CI 0.630to 0.886) respectively. As expected, AC and DC displayed good performance to discriminate recurrence-free subjects. The pointcuts of AC and DC were −5.1 and 4.8 ms respectively.

**Figure 3:**
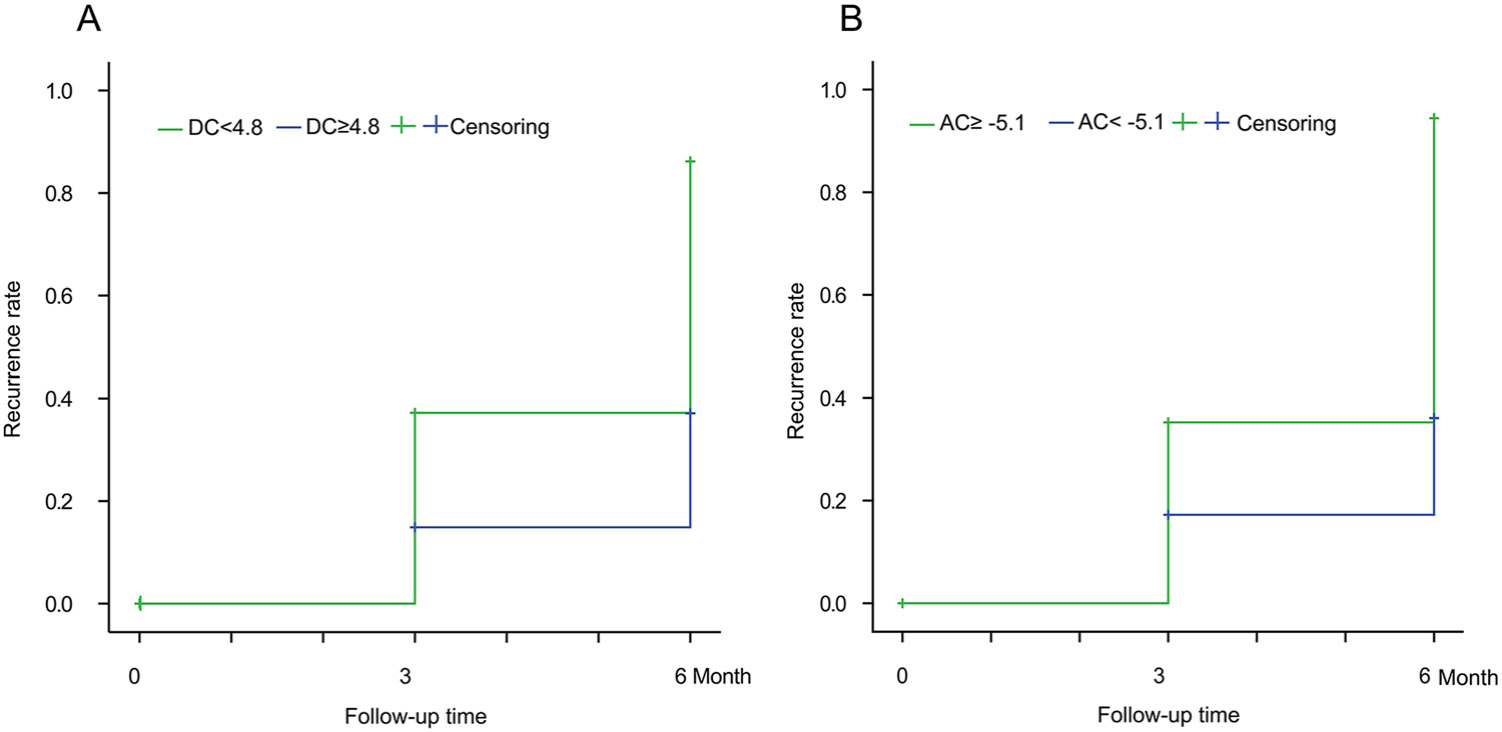
Kaplan–Meier analysis of AF recurrence-free rate according to AC and DC. A. Recurrence-free rates in 6 months follow-up of DC < 4.8 ms vs. DC ≥ 4.8 ms. B. Recurrence-free rate in 6 months follow-up of AC ≥ −5.1 ms vs. AC < −5.1 ms.

Next, the recurrence-free rates were analyzed using above cut off values. During 6 months of follow-up, 34 (22.1%) and 54 (35.1%) of the 154 patients showed AF recurrence within 3 and 6 months following up, respectively. Kaplan–Meier analysis according to DC (DC < 4.8 ms, log-rank, Chi-square = 159.149, P < 0.001; Fig. 3) showed significant benefit for clinical outcome after catheter ablation. Also, Kaplan–Meier analysis according to AC (AC ≥ -5.1 ms,log-rank, Chi-square = 163.907, P < 0.001; Fig. 3) showed significant high AF-free survival rate after catheter ablation.

## Discussion

In this report, a novel measure was adopted to explore the eletroneurophysiological properties in AF patients before and after ablation. Firstly, ablation significantly reduced the DCs both in recurrent and recurrence-free groups; the mean DC of recurrent group had recovered significantly 3 and 6 months after ablation; however, the mean DC of recurrence-free group kept at a relative low level through out the 6 months; the mean DCs were significantly lower in recurrence-free group than these in recurrent group at each time point. Secondly, ablation significantly increased the ACs both in recurrent and recurrence-free groups; the mean AC of recurrent group had dropped down significantly 3 and 6 months after ablation; impressively, the mean AC of recurrence-free group kept at a relative high level; the ACs were significantly higher in recurrence-free group than these in recurrent group at each time point. Thirdly, further Kaplan-Meier analysis showed that subjects with DC < 4.8 ms or AC ≥ −5.1 ms displayed significantly higher recurrence-free rates compared to subjects with DC ≥ 4.8 ms or AC < −5.1 ms. Finally, other HRV indices, such as AHR, SDNN, RMSSD and LF/HF were erratic in two groups before and after ablation. These results suggested that AC and DC displayed a relative superiority to early discriminate the recurrence-free and recurrent subjects.

As we known, to quantify and/or to distinguish between the cardiac sympathetic and parasympathetic neural modulations are hard problems^13,14^. Theoretically, the AC and DC could reflect the cardiac sympathetic and parasympathetic modulations on heart respectively^15–17^. Suppose above theory is correct; in our report; the significant decline of the absolute values of AC and DC three days after ablation suggests patients with AF were in a higher DC and lower AC state before ablation; alternatively; patients with AF were in a lower sympathetic and higher parasympathetic modulating state before ablation; this deduction is in line with some reports^18,19^. In addition, lower DC associated with higher AF-free rate after ablation suggest that vagal denervation and destroy of ectopic firing originating from the cardiomyocyte sleeves are critical for AF therapy^19^. While, higher AC should be a secondary phenomenon, that is, high sympathetic activity is most likely to be caused by vagal denervation, which weakened antagonism to sympathetic activity.

A recent study using a cardiovascular system model showed that the eletroneurophysiological properties of DC and AC were highly influenced by the timescales (*T*) and wavelet scales (*s*) used in the computation^20^. Either DC or AC was solely dependent on vagal activity under the scales of *T* = 1 and *s* = 2; however, with the scales of *T* = 3 and *s* = 5, both DC and AC were correlated positively to sympathetic activity and negatively to vagal activity. In our study, the DC and AC were computed with *T* = 1 and *s* = 2. If the above theory is adopted to interpret our results, high DC and low AC might predict more severe disorders of vagal modulation in heart eletroneurophysiology and then easily cause early AF recurrence.

The recurrence of AF might be caused by inadequate autonomic denervation or neuroplasticity^2,3,21^. In our study, 35.1% subjects displayed clear evidence of AF recurrence 3 months after CPVI. Since dormant conduction was assessed by isoproterenol infusion, pulmonary vein pacing, coronary vein and intra atrial pacing during CPVI, the early recurrence of AF is more likely to be caused by the ganglionic plexi renovation and/or PV-left atrial conductions recovery. Interestingly, AC and DC displayed a potential performance to discriminate the recurrence.

Our data showed that all the SDNN, RMSSD and LF/HF of recurrence-free and recurrence groups displayed a separation and coincidence tendency after the ablation, which might reflect the injury and repair of PV after ablation. Nevertheless, no stable tendency of the HRV parameters was found. A study examining 636 Chinese subjects without heart disease showed that DC, SDNN, RMSSD varied by age, gender and circadian rhythm^22^. DC decreased gradually with age increase in normal population, the average DCs of subjects aged 50–59 and 60–70 years old were 6.61 ± 1.38 and 6.18 ± 1.93 ms respectively. In our study, the average age of the subjects was 60.0 ± 10.1 years old, their average DC before ablation (7.15 ± 2.15 ms) was higher than above normal Chinese with similar age. This difference may also be explained by that AF associated with higher vagal tension^18,19^.

In our report, 85 (55.2%), 26 (16.9%) and 74 (48.1%) subjects were treated with amiodarone,propafenone and metoprolol, respectively. As we know, amiodarone slows conduction rate and prolongs the refractory period of the SA and AV nodes;^23^ propafenone slows the influx of sodium ions into the cardiac muscle cells, causing a decrease in excitability of the cells;^24^ and metoprolol blocks β1 adrenergic receptors in heart muscle cells, thereby decreasing the slope of phase 4 in the nodal action potential and prolonging repolarization of phase 3^25^. In general, these medicines are mainly to control arrhythmia by affecting the action potential of cardiac myocytes. DC and AC are derived from the heartbeat intervals. Thus, amiodarone, propafenone and metoprolol administration should have limited effect on AC and DC. Be the evidence, our data showed that none of the three medicines changed the HRV indexes or AC and DC significantly, which is also consistent with other reports partially^26,27^.

Since Kantelhardt JW *et al*. applied the PRSA algorithm in AC and DC of the heart rate^17^, more and more studies have found that AC and DC are related not only to physiological activity but also to a number of pathophysiological states. DC decreased gradually with age increase in normal population;^22^ the magnitudes of DC and AC increased progressively as breathing frequency decreased;^28^ lower DC of heart rate is a strong and independent predictor of 1-year mortality in patients with severe aortic stenosis undergoing transcatheter aortic valve implantation;^29^ AC is positively associated with heart failure grade in patients with dilated cardiomyopathy;^16^ lower DC assessed from short-term recordings is a strong and independent predictor of mortality and cardiovascular mortality after myocardial infarction^30^. These reports showed the complex role of ANS in the physiological and pathological process. More extensive and deeper researches are needed to figure out the overview of ANS.

There are several limitations in our study, firstly, we had only followed up the patients for only 6 months, we will continue to observe these patients and will provide long-term data in our next study; secondly; we computed the AC and DC under scales *T* = 1 and *s* = 2 only, we had not computed the AC/DC using other scales.
